# Alcohol intoxication and lack of helmet use are common in electric scooter-related traumatic brain injuries: a consecutive patient series from a tertiary university hospital

**DOI:** 10.1007/s00701-021-05098-2

**Published:** 2022-01-14

**Authors:** Eetu N. Suominen, Antti J. Sajanti, Eero A. Silver, Veerakaisa Koivunen, Anton S. Bondfolk, Janne Koskimäki, Antti J. Saarinen

**Affiliations:** 1 Department of Clinical Medicine, University of Turku, Turku, Finland; 2 Neurocenter, Department of Neurosurgery, Turku University Hospital and University of Turku, Turku, Finland; 3 Department of Anaesthesia and Intensive Care, University of Turku and Turku University Hospital, Turku, Finland; 4 Department of Paediatric Orthopaedic Surgery, University of Turku and Turku University Hospital, Kiinamyllynkatu 4-8, 20521 Turku, Finland; 5 Department of Orthopaedics and Traumatology, University of Helsinki and Helsinki University Hospital, Helsinki, Finland

**Keywords:** Traumatic brain injury, TBI, Electric scooter, Brain trauma, Traffic accident

## Abstract

**Purpose:**

Clinicians have increasingly encountered traumatic brain injuries (TBI) related to electric scooter (ES) accidents. In this study, we aim to identify the modifiable risk factors for ES-related TBIs.

**Methods:**

A retrospective cohort of consecutive patients treated for ES-related traumatic brain injuries in a tertiary university hospital between May 2019 and September 2021 was identified and employed for the study. The characteristics of the accidents along with the clinical and imaging findings of the injuries were collected from the patient charts.

**Results:**

During the study period, 104 TBIs related to ES accidents were identified. There was a high occurrence of accidents late at night and on Saturdays. In four cases, the patient’s helmet use was mentioned (3.8%). Seventy-four patients (71%) were intoxicated. At the scene of the accident, seventy-seven (74%) of the patients had a Glasgow Coma Scale score of 13–15, three patients (3%) had a score of 9–12, and two patients (2%) had a score of 3–8. The majority (83%) of TBIs were diagnosed as concussions. Eighteen patients had evidence of intracranial injuries in the imagining. Two patients required neurosurgical procedures. The estimated population standardized incidence increased from 7.0/100,000 (95% CI 3.5–11/100,000) in 2019 to 27/100,000 (95% CI 20–34/100,000) in 2021.

**Conclusions:**

Alcohol intoxication and the lack of a helmet were common in TBIs caused by ES accidents. Most of the accidents occurred late at night. Targeting these modifiable factors could decrease the incidence of ES-related TBIs.

**Supplementary Information:**

The online version contains supplementary material available at 10.1007/s00701-021-05098-2.

## Introduction

At the end of 2017, rental electric scooters (ESs) were first introduced into the USA as a new, nationwide means of transport. Recently, they have become a significant means of transportation for urban residents and have assumed a substantial portion of the micro-mobility market in cities worldwide.

Since the introduction of rentable ESs in Finland, there have been frequent reports of accidents in the media and a widespread debate over the legislation on ES usage. In our city, ESs have been available for rent since the beginning of May 2019. In Finland, the maximum speed of ESs is limited to 25 km per hour. In the middle of July 2021, the maximum speed was lowered to 15 km/hour during the night at weekends (Fri-Sun, from 23:00 to 05:00). By jurisdiction, ESs are treated in a similar way to bicycles concerning helmet use and intoxication. Helmets are required by law but not enforced. Driving under the influence of alcohol is forbidden but not strictly enforced unless the driver causes evident danger to the public.

The findings of a recent review suggest frequent injuries to the head and extremities in ES-related accidents [30]. The reported occurrence of head injuries has varied between 15 and 40% of all ES-related injuries [16, 31]. Since ESs have only recently been introduced to the public, up-to-date data on injury patterns of ES-related TBIs are sparse. As their national and worldwide popularity increases, it is critical to provide better evidence on the injury patterns and severity of ES-related brain traumas. This information enables estimating the burden these injuries pose to the emergency departments and the health care system. Understanding the risk factors associated with injuries may better inform public decisions, affect individual safety, and guide future policy.

In this study, we describe the characteristics of ES-related traumatic brain injuries treated in a tertiary university hospital over a 2.5-year period. We also describe modifiable risk factors for electric scooter injuries. We hypothesized that the rate of helmet usage is low, the accidents occur mainly late at night, and that the accidents are frequently involved with alcohol intoxication.

## Patients and methods

### Study design

This study was conducted in the city of Turku, located in Southwest Finland. Approximately 20% of the population in Turku is aged between 20 and 29 years [19]. Turku is the only city located in the region of Southwest Finland in which rentable ESs are available. Patients requiring immediate or urgent specialized care in the city of Turku can seek treatment in Turku University Hospital which is a tertiary hospital covering a city population of 194,391 in 2020. Our study center is the primary emergency department (ED) in the region, in which all traumatic injuries are treated. In 2020, a total of 1472 patients with TBIs were treated at our hospital. An institutional research board permit was obtained.

**Fig. 1:**
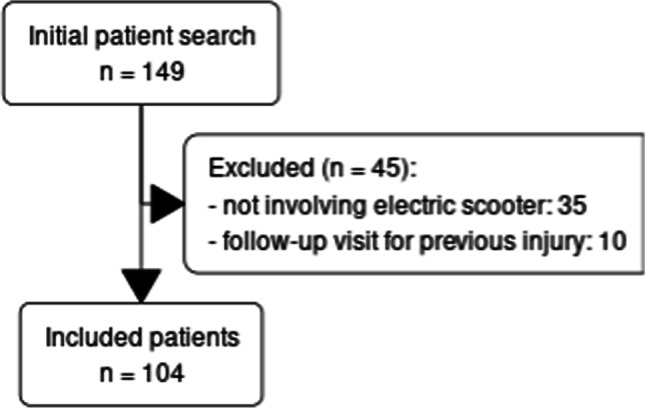
Flowchart of the patient selection. Injuries related to other transportation methods than electric scooters and follow-up visits for previous injuries were excluded

A retrospective cohort of consecutive patients was collected using patient charts from our center. We searched for patients diagnosed with intracranial injuries between 1st of May 2019 and 30th of September 2021 using International Classification of Diseases (ICD) codes S06.0-S06.9 and a keyword search for electrical scooters (electric scooter, E-scooter, etc.). After this, a keyword search without the ICD-codes was conducted for TBIs involving electrical scooters. This provided no additional patients. The involvement of an ES was then verified, and eligible patients were included in the study (Fig. 1). Patients with injuries related to non-electronic scooters, moped scooters, or other seated scooters were excluded. Follow-up visits for previous ES-related injuries were excluded.

### Data collection

The following data were collected: age, gender, time and date of the injury, helmet use, presence of primary unconsciousness, transportation by ambulance to the hospital, blood or breath alcohol level, Glasgow Coma Scale (GCS) score assessed at the scene of the accident or the emergency department (ED), possible other injuries sustained, the exact ICD-code, the type of radiological imaging, length of hospitalization, and interventions required for the TBI. Computed tomography (CT) and magnetic resonance imaging (MRI) scans were evaluated.

We classified the intracranial traumas according to the imaging findings: (1) loss of consciousness or concussion without CT or MRI findings (referred to as imaging negative), (2) CT or MRI diagnosed brain hemorrhages treated non-operatively (imaging positive), and (3) patients with CT and MRI diagnosed brain hemorrhages requiring operative treatment (imaging positive requiring operative treatment) [28]. The patients were also classified according to the Glasgow Coma Score addressed at the scene of the accident as follows: (1) mild TBI with GCS score 13–15, (2) moderate TBI with GCS score 9–12, and (3) severe TBI with GCS score 3–8.

### Data analysis

Analysis was conducted in R (R 4.1.1, R Core Team, 2020). Descriptive analyses were reported as the means and SDs, medians and both quartiles and ranges, or absolute numbers and percentages referring to the study groups. Age-standardized rate allows comparison of incidences between different populations [6]. We calculated direct age- and sex-standardized incidence rates by multiplying the age-specific and age-sex-specific incidence rates with the corresponding age bracket weights of the European standard population (ESP) [6]. The 95% confidence intervals for European age-standardized rates (EASR) were calculated using Poisson approximation [6].

## Results

### Patient and event characteristics

**Fig. 2:**
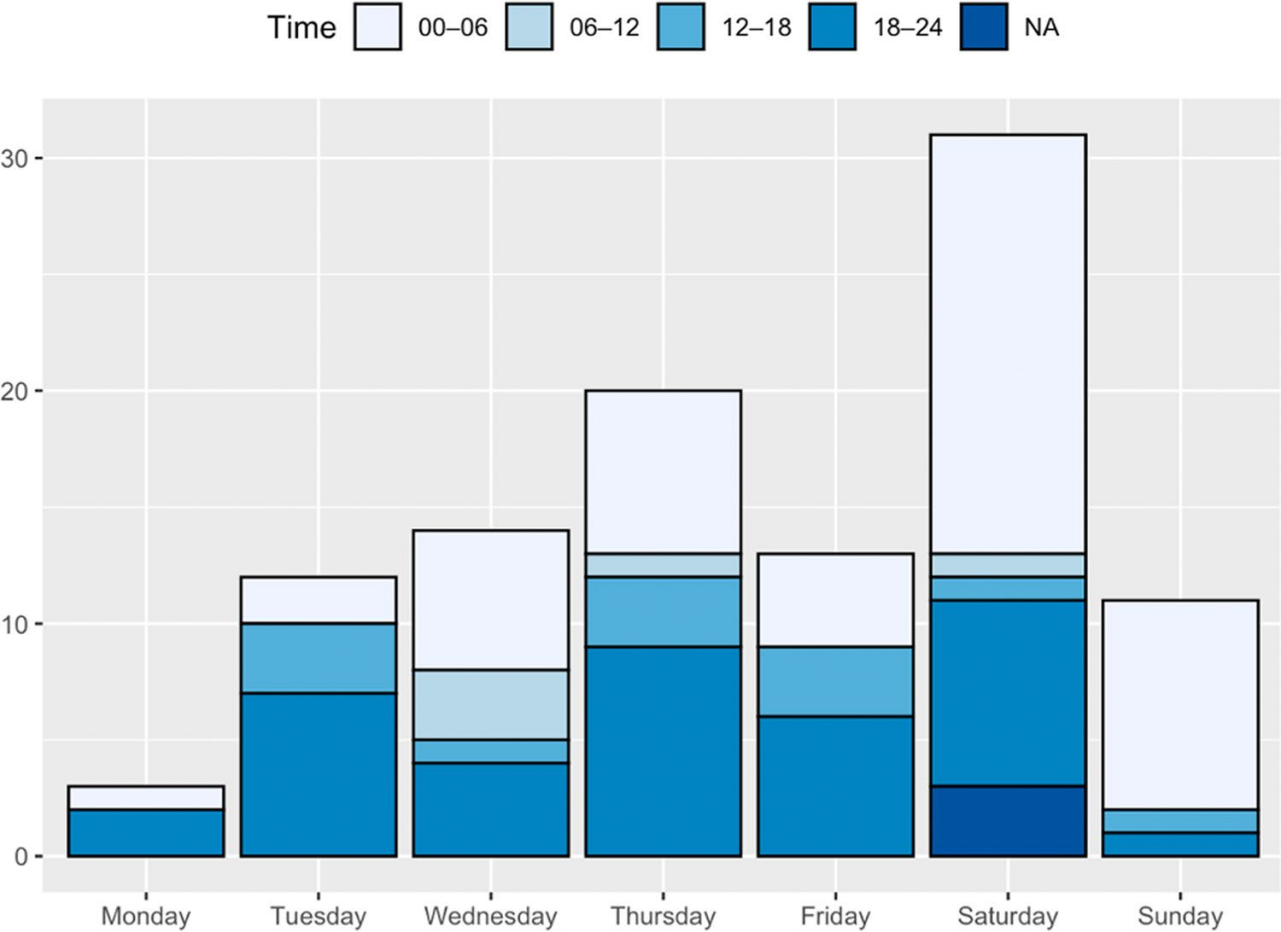
Daily prevalence and the time of the ES-related injuries. NA = Day known; exact time not available

**Fig. 3:**
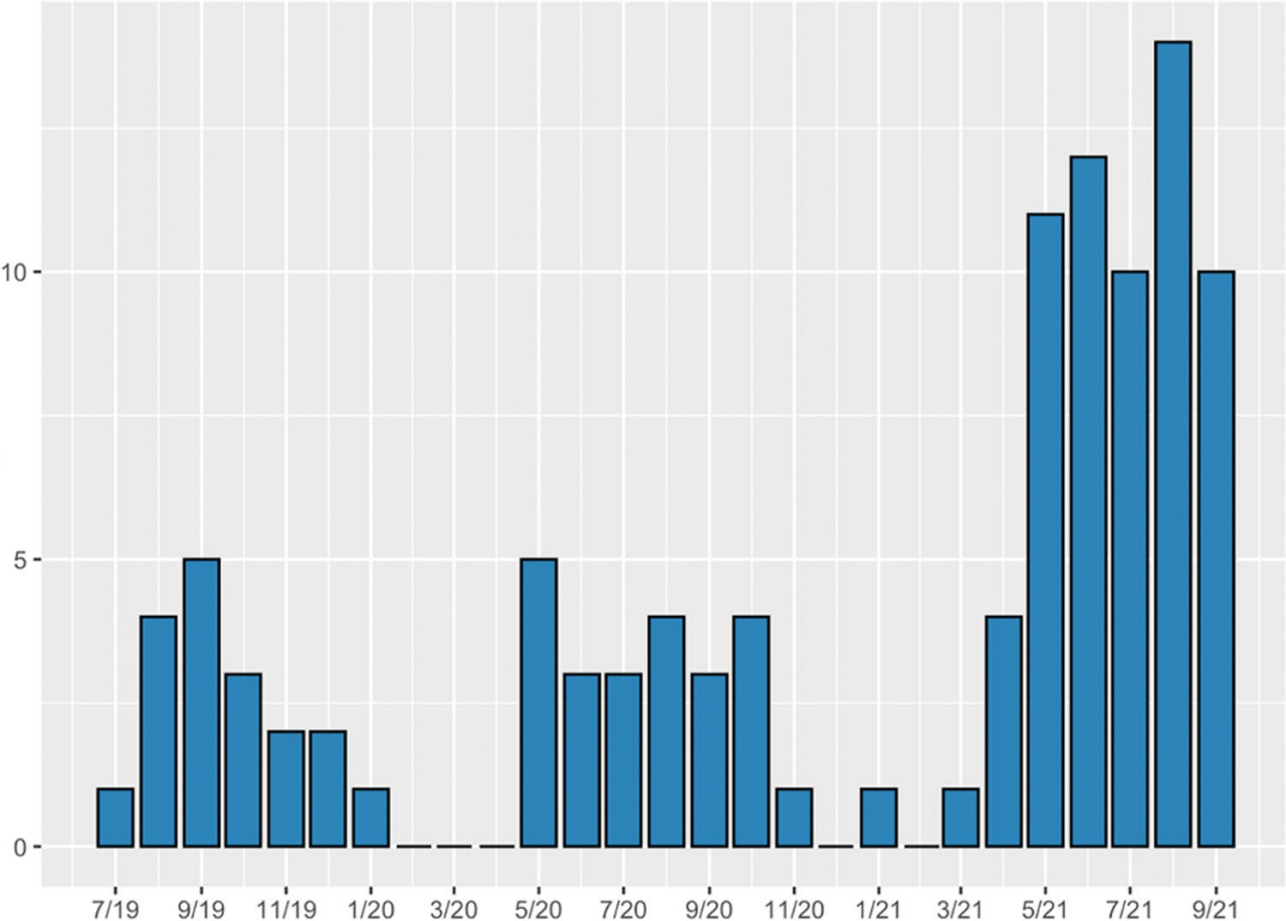
Monthly prevalence. The prevalence of ES-related TBIs increased towards the end of the study period and was lower in the winter months. Rentable ESs were generally not available during the snow cover

In total, 149 patients were identified, of whom after exclusion 104 were selected for the final analyses (Fig. 1). The demographic details of the patient population are presented in Table 1. There were 63 (61%) males and 41 (39%) females. The median age at the time of injury was 23.7 (range 5–71) years. Forty-nine percent of the patients were young adults, between the ages of 18 and 25. The highest prevalence was 215/100,000 in the age group of 20–25 years (264 in males and 174 in females, Table 2). The prevalence was higher in males (68 in males vs. 41 in females, Supplementary Tables I, II). The EASR during the whole study period was 44/100,000 (95%CI 35–53/100,000, Table 2). EASRs were 57/100,000 (95%CI 42–72/100,000) for males and 32/100,000 (95%CI 21–42/100,000) for females during the study period. There was an increase in the EASR from 7.0/100,00 in 2019 (95%CI 3.5–11/100,000) to 27/100,000 in 2021 (95%CI 20–34/100,000, Supplementary Tables III, V). There was a peak of injuries late at night: 81% of the accidents occurred between 6 p.m. and 6 a.m. (Fig. 2). The occurrence was the highest during Saturdays (29.8%) and Thursdays (19.2%). The injuries were most common during the summer months: 82% of the accidents occurred between May and September (Fig. 3).

**Table 1:** Patient characteristics

Age, median (years), (Q1, Q3, range)	
All	24 (20.5, 30.1, 5–70)
Male	24 (21.1, 32.8, 8–55)
Female	22 (20.3, 26.8, 5–70)
**Age group (years), n (%)**	
< 18	10 (9.6)
18–25	51 (49.0)
25–40	31 (29.8)
> 40	12 (11.5)
**Sex**	
Male	63 (60.6)
Female	41 (39.4)
**Time of accident**	
Midnight to 6 a.m	47 (45.2)
6 a.m. to noon	5 (4.8)
Noon to 6 p.m	12 (11.5)
6 p.m. to midnight	37 (35.6)
Unknown	3 (2.9)
**Day of accident**	
Monday	3 (2.9)
Tuesday	12 (11.5)
Wednesday	14 (13.5)
Thursday	20 (19.2)
Friday	13 (12.5)
Saturday	31 (29.8)
Sunday	11 (10.6)
**Month of accident**	
January	1 (1.0)
February	0 (0)
March	1 (1.0)
April	4 (3.8)
May	16 (15.4)
June	15 (14.4)
July	14 (13.5)
August	22 (21.2)
September	18 (17.3)
October	7 (6.7)
November	4 (3.8)
December	2 (1.9)
**Intoxication**	
No intoxication	21 (20.2)
Alcohol reported	74 (71.2)
< 0.5‰	1 (1.4)
0.5–1.2‰	7 (9.6)
> 1.2‰	58 (79.4)
Alcohol reported but level unknown	7 (9.6)
Unknown	9 (8.7)
**Helmet use**	
Yes	4 (3.8)
No	67 (64.4)
Unknown	33 (31.7)
**Collision**	
No	96 (92.3)
Car	5 (4.8)
E-scooter	1 (1.0)
Bicycle	1 (1.0)
Moped	1 (1.0)

**Table 2:** European age standardized rates of all 321 ES-related TBIs in Turku 2019–2021

Age	European standard population 2013	Population in Turku (2020)	ES-related TBIs	Prevalence in the age group per 100,000	European age standardized rate (EASR)
0–4	5,500	8,014	0	0.0	0.0
5–9	5,500	8,372	2	23.9	1.3
10–14	5,500	8,052	7	86.9	4.8
15–19	6,000	8,775	9	102.6	5.6
20–24	6,000	19,995	43	215.1	12.9
25–29	6,500	19,069	17	89.2	5.4
30–34	7,000	15,181	9	59.3	3.9
35–39	7,000	13,266	5	37.7	2.6
40–44	7,000	11,419	1	8.8	0.6
45–49	7,000	9,781	2	20.5	1.4
50–54	6,500	10,578	7	66.2	4.6
55–59	6,000	10,809	1	9.3	0.60
60–64	5,500	10,435	0	0.0	0.0
65–69	5,000	10,560	0	0.0	0.0
70–74	9,000	11,317	1	8.8	0.44
75– > 90	5,500	18,768	0	0.0	0.0
**All ages**	**100,000**	**194,391**	**104**	**53.5**	**44.2**

The majority of the patients (64%) did not wear a helmet at the time of the accident and in only four cases was the patient’s use of a helmet mentioned (3.8%). No information concerning helmet usage was available for 33 patients (32%). Ten cases involved riding the scooter with two persons onboard (9.6%). Primary unconsciousness was reported in 33 (32%) patients. GCS addressed at the scene of the accident was 13–15 for seventy-seven (74%) patients, 9–12 for three (3%) patients, and 3–8 for two (2%) patients. In 22% of the cases, GCS was not reported. Seventy-four patients (71%) were brought to ED by ambulance. Forty-nine patients were discharged directly from ED without head imaging or imaging findings. In patients requiring surveillance, 36 were discharged during the first 24 h. Fourteen patients required surveillance for one to seven days (mean 2.0, range 1–). Three patients required longer surveillance and were discharged after seven, eight, and 14 days. None of the patients had ongoing antithrombotic medication.

In total, 74% of the patients were under the influence of alcohol. The mean alcohol level in breath or blood was 1.8‰ (range, 0.3–3.6) in intoxicated patients. Fifty-eight (77%) intoxicated patients had alcohol concentrations higher than 1.2‰. As mentioned, 81% of the accidents occurred between 6 p.m. and 6 a.m. In these cases, 86% of the patient were under the influence of alcohol. In one patient, there was a report of cannabis use in addition to alcohol intoxication.

Ten patients had a cranial fracture, nine had a maxillofacial fracture, and three had both skull and maxillofacial fractures. Two patients required operative treatment for maxillofacial fractures. Other fractures involved scapula, proximal phalange, and rib fractures.

### Traumatic brain injuries (TBIs)

A CT scan was performed on 80 patients and an MRI scan on two patients. Twenty-two patients (21%) were clinically evaluated and required no imaging. Most injuries were diagnosed as concussions (86 cases, 82%, Table 3). In patients with CT or MRI scans, 64 patients (78%) had no traumatic intracranial findings. Eighteen patients had imaging positive intracranial injury (Table 4). Two of those patients required neurosurgical procedures. Imaging positive injuries were as follows: diffuse traumatic brain injury in three patients (3%), brain contusion in seven patients (7%), epidural hemorrhage in one patient (1%), traumatic subdural hemorrhage in seven patients (7%), and traumatic subarachnoid hemorrhage in seven patients (7%). One patient required hematoma evacuation after which an intracranial pressure monitoring was installed. The patient remained under surveillance in the neurosurgical department for two days. Another patient required intracranial pressure monitoring and remained under surveillance for seven days. Eighty-nine percent of the imaging positive patients were under the influence of alcohol (mean alcohol level 1.8‰.).

**Table 3:** Injury characteristics

	*n* (%)
**Transport to emergency department**	
Ambulance	74 (71.2)
Ambulatory	30 (28.8)
**GCS score at the scene of the accident**	
13–15	77 (74.0)
9–12	3 (2.9)
3–8	2 (1.9)
Unknown	22 (21.2)
**Primary unconsciousness**	
Yes	33 (31.7)
No	54 (51.9)
Unknown	17 (16.3)
**Traumatic brain injuries (ICD-10)**	
Concussion (S06.0)	86 (82.7)
Diffuse traumatic brain injury (S06.2)	3 (2.9)
Brain contusion (S06.3)	7 (6.7)
Epidural hemorrhage (S06.4)	1 (1.0)
Traumatic subdural hemorrhage (S06.5)	7 (6.7)
Traumatic subarachnoid hemorrhage (S06.6)	7 (6.7)
**Cranial fractures**	
Yes	22 (21.2)
Cranial	10 (45.5)
Maxillofacial	9 (40.9)
Both	3 (13.6)
No	82 (78.8)
**Other fractures**	
Yes	3 (2.9)
Upper extremity	2 (66.6)
Rib	1 (33.3)
No	101 (97.1)
**Radiological imaging**	
Yes	82 (78.8)
CT	80 (97.6)
MRI	2 (2.4)
No	22 (21.2)
**Imaging**	
Imaging negative	64 (78.0)
Imaging positive	16 (19.5)
Imaging positive requiring neurosurgical treatment	2 (2.4)
**Length of hospital stay**	
< 4 h	49 (47.1)
4–24 h	38 (36.5)
1–7 days	14 (13.5)
> 7 days	3 (2.9)

*GCS*, Glasgow Coma Scale

**Table 4:** Characteristics of imaging positive patients

Gender	Age	Time of the incident	Type of accident	Alcohol level	Reported helmet use	Primary GCS*	Primary unconsciousness	Fracture	Transportation to the ED	Imaging	Description of the findings	Operative treatment	Length of hospital stay
Male	55	18–24	Fall	2.2	No	13–15	Yes	Maxillofacial	Ambulance	CT	Traumatic subarachnoid hemorrhage of 2.8 mm and brain contusion	No	6 h
Male	38	00–06	Fall	2.9	No	13–15	No	Cranial	Ambulance	CT	CT Brain contusion of 0.97 ml	No	2 days
Male	33	18–24	Fall	3.6	No	13–15	Yes	Cranial	Ambulance	CT	Traumatic subdural hemorrhage and brain contusion	No	2 days
Female	44	00–06	Fall	1.1	No	13–15	No	Maxillofacial	Ambulance	CT	Diffuse brain injury	No	8 days
Male	22	00–06	Fall	1.1	Unknown	13–15	Yes	Cranial	Ambulance	CT	Traumatic subarachnoid hemorrhage	No	2 days
Male	39	06–12	Fall	2.0	No	13–15	Unknown	None	Ambulance	CT	Traumatic subarachnoid hemorrhage and brain contusion	No	Until morning
Female	20	00–06	Fall	2.4	No	3–8	Yes	Cranial	Ambulance	CT	Traumatic subdural hemorrhage and traumatic subarachnoid hemorrhage of 11 mm	Intracranial pressure monitoring	7 days
Female	20	00–06	Fall	1.7	No	9–12	Unknown	None	Ambulance	MRI	Diffuse brain injury	No	Until morning
Male	21	18–24	Fall	0	Unknown	13–15	Yes	Cranial	Ambulatory	CT	Brain contusion of 14 ml	No	Until morning
Male	27	00–06	Fall	1.8	No	13–15	Yes	Cranial	Ambulance	CT	Traumatic subdural hemorrhage of 5.9 mm	No	2 days
Male	14	18–24	Collision with a motorbike	0	No	9–12	Yes	Cranial	Ambulance	CT	Traumatic subdural hemorrhage of 8.0 mm	No	14 days
Male	24	00–06	Fall	2.0	No	14	Yes	Maxillofacial and Cranial	Ambulance	CT	Traumatic subdural hemorrhage of 6.0 mm, traumatic subarachnoid hemorrhage, and brain contusion	No	2 days
Male	23	00–06	Fall	2.7	Unknown	9	Yes	None	Ambulance	CT	Traumatic subarachnoid hemorrhage	No	2 days
Male	48	00–06	Fall	2.2	No	15	No	Maxillofacial and cranial	Ambulance	CT	Traumatic subarachnoid hemorrhage	Hematoma evacuation and intracranial pressure monitoring	2 days
Female	19	00–06	Fall	1.5	No	5	Yes	None	Ambulance	CT	Traumatic subarachnoid hemorrhage	No	2 days
Male	50	18–24	Fall	2.8	No	13–15	Yes	Maxillofacial and cranial	Ambulance	CT	Traumatic subarachnoid hemorrhage	No	4 days
Male	19	00–06	Fall	1.7	No	13–15	Unknown	Maxillofacial	Ambulance	CT and MRI	Traumatic subarachnoid hemorrhage	Surgical treatment of maxillofacial fractures	2 days
Male	48	18–24	Fall	1.5	No	13–15	No	Rib	Ambulance	CT	Traumatic subarachnoid hemorrhage	Surgical treatment for fractured scapula	1 day

^*^Glasgow Coma Scale, exact score if known

## Discussion

The present study demonstrates an evident increase in the incidence of ES-related TBIs in our institute between 2019 and 2021. The accident patterns were as we hypothesized: high alcohol levels and lack of helmet use were prominently present in patients with ES-related TBIs. Moreover, most accidents occurred during the summer months and late at night.

Common ES-related injuries include head traumas, TBIs, and fractures [18]. Previous studies have reported a high incidence of head injuries and TBIs in ES accidents [11, 20, 26, 32]. Head traumas, half of which included TBI, were the most common reason for ED visits for ES injuries in a study of 70,544 cases [7]. A recent case series described 13 severe injuries, which required neurosurgical intervention including skull fractures, central cord syndrome, and vertebral compression fractures related to ES injuries [23]. In that study, one patient was pronounced dead on arrival due to epidural hematoma resulting in midline shift and bilateral frontal subarachnoid hemorrhages [23]. In our study, eighteen patients had imaging positive injuries with two patients requiring neurosurgical procedures. Most patients in this study were under the age of 30 years. TBIs are a major cause of death and disability in the younger adult population [4, 12, 14]. Although the long-term effects were not assessed in our study, previous results show that young patients can be vulnerable to long-term difficulties due to a lack of coping skills [5]. Both upper and lower extremity fractures requiring operative treatment are reported in ES-related accidents [8, 24, 29]. In our cohort, the low incidence of upper extremity fractures may indicate that these patients may not have been able to react to the sudden fall or collision.

Although ES-related injuries make up only a minor portion of all ED visits for traumatic injuries, the increasing trend in the current study and previous studies is alarming [7, 9, 17]. A recent study showed a twofold increase in the incidence of E-scooter-related injuries in the USA between 2018 and 2019 [7]. According to a recent study, the incidence of ES-related injuries increased from 2.42 in 2017 to 8.63 per 100,000 person-years in the USA [9]. As ES usage is becoming increasingly popular, a parallel increase would be expected to occur in the rates of ES-related accidents and TBIs. There was an increasing trend of cases towards the end of the study period, which is likely related to the increased number of ESs and the loosening of the Covid-19 pandemic restrictions. We presume that the ES-related incident numbers in our city could have been higher without the Covid-19 restrictions limiting bar and nightclub visits. This was supported by the substantial increase in accidents in the latter months of the study during which the Covid-19 restrictions were relaxed. Lowering the maximum nighttime speed of the ESs in July 2021 did not seem to reduce the incidence of TBIs in our study. We did not access the incidence of other ways of micro-transportation during the study period. Previous studies have compared ES and bicycle accidents. Studies show that the estimated ED presentation rates per million miles traveled citywide were higher among e-scooter riders than cyclists (RR 3.76; 95% CI, 3.08–4.59) and the ES riders are far less likely to wear helmets [3]. Trip purposes also vary, as bicycles are mainly used to commute to/from work and ESs are mainly used for social reasons and personal business [3, 10]. Cyclists are more frequently injured on the road in incidents involving motor vehicles, and e-scooter riders sustain more injuries on sidewalks without the involvement of other road users. Compared to bicycle accidents, e-scooter accidents more commonly occur on weekends and in association with alcohol [10].

Based on previous research, ES-related accidents are more frequent when compared to cycling or pedestrian accidents [31]. Similar to the findings of the present study, typical ES accidents occur in the summertime, late at night during the end of the week [26, 32]. Conditions such as cold weather, snow, and pandemic quarantine restrictions have been associated with lower rates of ES accidents [26, 32].

Electric scooter accidents are predominantly reported to involve only the scooter rider [2, 25, 26, 31]. In the current study, 92% of cases did not involve other road users, which indicates that most of the accidents result from handling errors. Furthermore, in 90% of the cases, there was a single person on board. Most ES-related injuries were related to alcohol intoxication in previous studies [7, 10, 20, 22, 31]. Our study showed a similar pattern, with 71% of the accidents involving alcohol intoxication. Noticeably, in alcohol-related accidents, the majority (79%) of patients had a breath or blood alcohol content higher than 1.2‰. In Finland, a law prohibits serving alcohol after 4 am, which forces bars and nightclubs to close and appears to demonstrate a significant contrast in the injury rates between 0–6 a.m. and 6–12 a.m. Alcohol intoxication is associated with an elevated risk for TBIs [13, 27]. A fivefold risk for TBI was observed in intoxicated patients in a previous study on ES-related injuries [32]. Another significant factor offering protection from TBIs is helmet use, which reduces the risk of serious head and facial injuries [21]. The helmet usage in ES-related accidents is strikingly low [1, 10, 15, 20, 30]. Supportive to our findings of reported helmet use (3.8%), data from 16 studies (*n* = 1656) showed that only 4.5% of cases of ES accidents involved helmet use [30]. The use of helmets might have been under-reported in our study, as the data on helmet use was missing in 33% of the cases.

### Limitations

This study was limited by the retrospective study design. There may have been patients with ES-related TBIs without proper diagnose codes and/or description of the accident in the patient charts. We did not assess the long-term outcomes of the injuries. Primary imaging findings, GCS on arrival, and the need for operative management can be used for assessing the potential severity of the TBIs; however, using these markers alone does not justify any conclusions on how severe or mild a TBI will be in a follow-up [28]. This was a consecutive patient series treated in a single hospital during a 2.5-year period. We did not assess the prevalence of TBIs related to other transportation methods. We do not have information on the rate of use and numbers of rentable ESs in our area.

## Conclusions

Our results show a high rate of alcohol intoxication and lack of helmet use in patients with brain injuries related to electric scooters. The incidence increased towards the end of the study period. Imaging positive injuries were present in 18 patients. These results may be used to inform the public and legislators about the risk of traumatic brain injuries related to electric scooter accidents. We hypothesize that targeting the modifiable factors such as helmet use, preventing driving while intoxicated, and restricting nighttime use could decrease ES-related brain injuries.

## Supplementary Information

Below is the link to the electronic supplementary material.

Supplementary file1 (DOCX 29 KB)

## Abbreviations

CT: Computer tomography
EASR: European age-standardized rates
ED: Emergency department
ES: Electric scooter
ESP: European Standard Population
GCS: Glasgow Coma Scale
MRI: Magnetic resonance imaging
TBI: Traumatic brain injury
