# Supplementary material for: Alcohol intoxication and lack of helmet use are common in electric scooter-related traumatic brain injuries: a consecutive patient series from a tertiary university hospital
Source: Acta Neurochir (Wien). 2022 Jan 14;164(3):643–53. doi: 10.1007/s00701-021-05098-2 (PMC8759433; doi:10.1007/s00701-021-05098-2)
Supplement: Supplementary file 1 — Supplementary file1 (DOCX 29 KB) [file 701_2021_5098_MOESM1_ESM.docx]

**Calculations**

European age–standardized rate in an age group = $\frac{Sp x ASR}{P}$, EASR in the population = $\sum_{n} (\frac{Sp x ASR}{P})$

| S_p_ = European Standard Population (ESP) in sex/age group |  |  |  |
| --- | --- | --- | --- |
| ASR = Age specific rate (cases per 100,000 persons) in sex/age group | | | |
| P = Population number in age/sex study group  n = number of age/sex groups  Confidence intervals  95% CI = EASR ± 1.96 x $\frac{EASR}{\surd\sum Cp}$  EASR = European age–standardized rate  C_p_ = cases in the study population | | |  |

**S1. European age–sex standardized rates of ES–related TBIs in males in Turku 2019–2021.**

| **Age** | **European Standard Population 2013** | **Male population in Turku (2020)** | **ES–related TBI Cases** | **Prevalence in the age group per 100,000** | **European Age Standardized Rate (EASR)** |
| --- | --- | --- | --- | --- | --- |
| 0–4 | 5,000 | 4,104 | 0 | 0.0 | 0.0 |
| 5–9 | 5,500 | 4,307 | 1 | 23.2 | 1.3 |
| 10–14 | 5,500 | 4,082 | 5 | 122.5 | 6.7 |
| 15–19 | 5,500 | 4,249 | 5 | 117.7 | 6.5 |
| 20–24 | 6,000 | 9,094 | 24 | 263.9 | 15.8 |
| 25–29 | 6,000 | 9,721 | 10 | 102.9 | 6.2 |
| 30–34 | 6,500 | 8,014 | 5 | 62.4 | 4.1 |
| 35–39 | 7,000 | 6,908 | 3 | 43.4 | 3.0 |
| 40–44 | 7,000 | 5,824 | 0 | 0.0 | 0.0 |
| 45–49 | 7,000 | 5,059 | 2 | 39.5 | 2.8 |
| 50–54 | 7,000 | 5,180 | 7 | 135.1 | 9.5 |
| 55–59 | 6,500 | 5,204 | 1 | 19.2 | 1.3 |
| 60–>90 | 25,500 | 21,365 | 0 | 0.0 | 0.0 |
| **All ages** | **100,000** | **93,111** | **63** | **67.7** | **57.1** |

**S2. European age–sex standardized rates of ES–related TBIs in females in Turku 2019–2021.**

| **Age** | **European Standard Population 2013** | **Female population in Turku (2020)** | **ES–related TBI Cases** | **Prevalence in the age group per 100,000** | **European Age Standardized Rate (EASR)** |
| --- | --- | --- | --- | --- | --- |
| 0–4 | 5,000 | 3,910 | 0 | 0.0 | 0.0 |
| 5–9 | 5,500 | 4,065 | 1 | 24.6 | 1.4 |
| 10–14 | 5,500 | 3,970 | 2 | 50.4 | 2.8 |
| 15–19 | 5,500 | 4,526 | 4 | 88.4 | 4.9 |
| 20–24 | 6,000 | 10,941 | 19 | 173.7 | 10.4 |
| 25–29 | 6,000 | 9,348 | 7 | 74.9 | 4.5 |
| 30–34 | 6,500 | 7,167 | 4 | 55.8 | 3.6 |
| 35–39 | 7,000 | 6,358 | 2 | 31.5 | 2.2 |
| 40–44 | 7,000 | 5,595 | 1 | 17.9 | 1.3 |
| 45–49 | 7,000 | 4,722 | 0 | 0.0 | 0.0 |
| 50–54 | 7,000 | 5,398 | 0 | 0.0 | 0.0 |
| 55–59 | 6,500 | 5,605 | 0 | 0.0 | 0.0 |
| 60–64 | 6,000 | 5,574 | 0 | 0.0 | 0.0 |
| 65–69 | 5,500 | 5,894 | 0 | 0.0 | 0.0 |
| 70–74 | 5,000 | 6,412 | 1 | 15.6 | 0.8 |
| 75–>90 | 9,000 | 11,835 | 0 | 0.0 | 0.0 |
| **All ages** | **100,000** | **101,320** | **41** | **40.5** | **31.8** |

**S3. European Age Standardized Rates of all ES–related TBIs in Turku in May–December 2019.**

| **Age** | **European Standard Population 2013** | **Population in Turku (2019)** | **ES–related TBI Cases** | **Prevalence in the age group per 100,000** | **European Age Standardized Rate (EASR)** |
| --- | --- | --- | --- | --- | --- |
| 0–4 | 5,000 | 8,291 | 0 | 0.0 | 0.0 |
| 5–9 | 5,500 | 8,383 | 0 | 0.0 | 0.0 |
| 10–14 | 5,500 | 7,803 | 0 | 0.0 | 0.0 |
| 15–19 | 5,500 | 8,848 | 2 | 22.6 | 1.2 |
| 20–24 | 6,000 | 19,662 | 8 | 40.7 | 2.4 |
| 25–29 | 6,000 | 19,135 | 2 | 10.5 | 0.6 |
| 30–34 | 6,500 | 14,808 | 2 | 13.5 | 0.9 |
| 35–39 | 7,000 | 13,025 | 1 | 7.7 | 0.5 |
| 40–44 | 7,000 | 11,356 | 1 | 8.8 | 0.6 |
| 45–49 | 7,000 | 9,615 | 0 | 0.0 | 0.0 |
| 50–54 | 7,000 | 10,882 | 1 | 9.2 | 0.6 |
| 55–>90 | 32,000 | 61,154 | 0 | 0.0 | 0.0 |
| **All ages** | **100,000** | **192,962** | **17** | **8.8** | **7.0** |

**S4. European Age Standardized Rates of all ES–related TBIs in Turku in 2020.**

| **Age** | **European Standard Population 2013** | **Population in Turku (2020)** | **ES–related TBI Cases** | **Prevalence in the age group per 100,000** | **European Age Standardized Rate (EASR)** |
| --- | --- | --- | --- | --- | --- |
| 0–4 | 5,000 | 8,014 | 0 | 0.0 | 0.0 |
| 5–9 | 5,500 | 8,372 | 1 | 11.9 | 0.7 |
| 10–14 | 5,500 | 8,052 | 1 | 12.4 | 0.7 |
| 15–19 | 5,500 | 8,775 | 3 | 34.2 | 1.9 |
| 20–24 | 6,000 | 19,995 | 9 | 45.0 | 2.7 |
| 25–29 | 6,000 | 19,069 | 5 | 26.2 | 1.6 |
| 30–34 | 6,500 | 15,181 | 2 | 13.2 | 0.9 |
| 35–39 | 7,000 | 13,266 | 1 | 7.5 | 0.5 |
| 40–44 | 7,000 | 11,419 | 0 | 0.0 | 0.0 |
| 45–49 | 7,000 | 9,781 | 0 | 0.0 | 0.0 |
| 50–54 | 7,000 | 10,578 | 1 | 9.5 | 0.7 |
| 55–59 | 6,500 | 10,809 | 1 | 9.3 | 0.6 |
| 60–>90 | 25,500 | 41,576 | 0 | 0.0 | 0.0 |
| **All ages** | **100,000** | **194,391** | **24** | **12.4** | **10.1** |

**S5. European Age Standardized Rates of all ES–related TBIs in Turku in January – September 2021.**

| **Age** | **European Standard Population 2013** | **Population in Turku (2020)** | **ES–related TBI Cases** | **Prevalence in the age group per 100,000** | **European Age Standardized Rate (EASR)** |
| --- | --- | --- | --- | --- | --- |
| 0–4 | 5,000 | 8,014 | 0 | 0.0 | 0.0 |
| 5–9 | 5,500 | 8,372 | 1 | 11.9 | 0.7 |
| 10–14 | 5,500 | 8,052 | 6 | 74.5 | 4.1 |
| 15–19 | 5,500 | 8,775 | 4 | 45.6 | 2.5 |
| 20–24 | 6,000 | 19,995 | 26 | 130.0 | 7.8 |
| 25–29 | 6,000 | 19,069 | 10 | 52.4 | 3.2 |
| 30–34 | 6,500 | 15,181 | 5 | 32.9 | 2.1 |
| 35–39 | 7,000 | 13,266 | 3 | 22.6 | 1.6 |
| 40–44 | 7,000 | 11,419 | 0 | 0.0 | 0.0 |
| 45–49 | 7,000 | 9,781 | 2 | 20.5 | 1.4 |
| 50–54 | 7,000 | 10,578 | 5 | 47.3 | 3.3 |
| 55–59 | 6,500 | 10,809 | 0 | 0.0 | 0.0 |
| 60–64 | 6,000 | 10,435 | 0 | 0.0 | 0.0 |
| 65–69 | 5,500 | 10,560 | 0 | 0.0 | 0.0 |
| 70–74 | 5,000 | 11,317 | 1 | 8.8 | 0.4 |
| 75–>90 | 9,000 | 18,768 | 0 | 0.0 | 0.0 |
| **All ages** | **100,000** | **194,391** | **63** | **32.4** | **27.1** |
